# Efficacy of minimally invasive surgery for the treatment of hypertensive intracerebral hemorrhage: A protocol of randomized controlled trial

**DOI:** 10.1097/MD.0000000000024213

**Published:** 2021-01-22

**Authors:** Jiang Liu, Jing Cheng, Hongjun Zhou, Chunyan Deng, Zhengxin Wang

**Affiliations:** Center for Neurological Diseases, The Third Affiliated Hospital of Chongqing Medical University, Chongqing 401120, China.

**Keywords:** hypertensive intracerebral hemorrhage, medication treatment, minimally invasive surgery, protocol, reduction ;

## Abstract

**Introduction:**

Hypertensive intracerebral hemorrhage (HICH) is the most serious complication of hypertension. Clearing intracranial hematoma as soon as possible, reducing brain cell edema, and controlling intracranial pressure could effectively reduce neuron damage, lower patient mortality, and improve patient prognosis. At present, minimally invasive surgery (MIS) has been widely used and plays an important role in the treatment of HICH. However, it is still in controversies about the choice of surgical treatment and medication treatment for HICH. Therefore, we try to conduct a randomized, controlled, prospective trial to observe the efficacy of MIS treatment against HICH compared with medication treatment.

**Methods:**

Patients will be randomly divided into treatment group and control group in a 1:1 ratio using the random number generator in Microsoft Excel. Stereotactic soft channel minimally invasive intracranial hematoma puncture and drainage treatment and medication treatment will be applied respectively. The outcomes of intracerebral hemorrhage volume, Glasgow coma scale, National Institutes of Health Stroke Scale will be recorded.

**Conclusions:**

The findings of the study will be helpful for the choice of MIS and conservative treatment when treating HICH patients.

**Trial registration:**

OSF Registration number: DOI 10.17605/OSF.IO/ME6Y5.

## 1 Introduction

Hypertensive intracerebral hemorrhage (HICH) is the most serious complication of hypertension, which frequently occurs in middle-aged male, especially in winter and spring. It is caused by the weakening blood vessel due to the damage of small arteries in the brain caused by long-term hypertension.^[1,2]^ HICH is a serious cerebrovascular disease which affects about 4 million people worldwide each year with high mortality and disability rate.^[3]^ Clearing intracranial hematoma as soon as possible, reducing brain cell edema, and controlling intracranial pressure could effectively reduce neuron damage, lower patient mortality, and improve patient prognosis.^[4,5]^

The bleeding volume in most patients with HICH is 20 to 40 mL, which is a moderate amount. For those patients, either medication treatment or surgical treatment could be applied. Medication treatment for HICH is the most basic therapeutic choice, including controlling blood pressure and glucose, anti-infection, relieving cerebral edema, fluid infusion, nutritional support, and symptomatic treatment.^[6]^ It shows certain curative effects, but it may also prolong the hematoma compression period to damage brain tissue, which could possibly result in serious neurological deficit. Theoretically, surgery has the potential to rapidly remove the hematoma to reduce brain damage. At present, minimally invasive surgery (MIS) has been widely used and plays an important role in the treatment of HICH. The minimally invasive hematoma evacuation is a simple and quick operation, and it could avoid the injury of brain tissues and blood vessels to reducing the risk of postoperative mortality and complications.^[4,7,8]^

However, it is still in controversies about the choice between surgical treatment and medication treatment for HICH.^[9]^ Although many studies showed that MIS is significantly better than medication treatment,^[10]^ many studies believe that different inclusion criteria could affect the results, and most patients in the surgical treatment group are more severe than those in the medication treatment group, which could also affects the conclusions.^[11]^ In addition, several randomized controlled trials have failed to demonstrate improvement in outcomes of patients in surgical treatment.^[12]^ Therefore, we try to conduct a randomized, controlled, prospective trial to observe the efficacy of MIS treatment against HICH compared with medication treatment.

## 2 Methods

### 2.1 Study design

**Figure 1:**
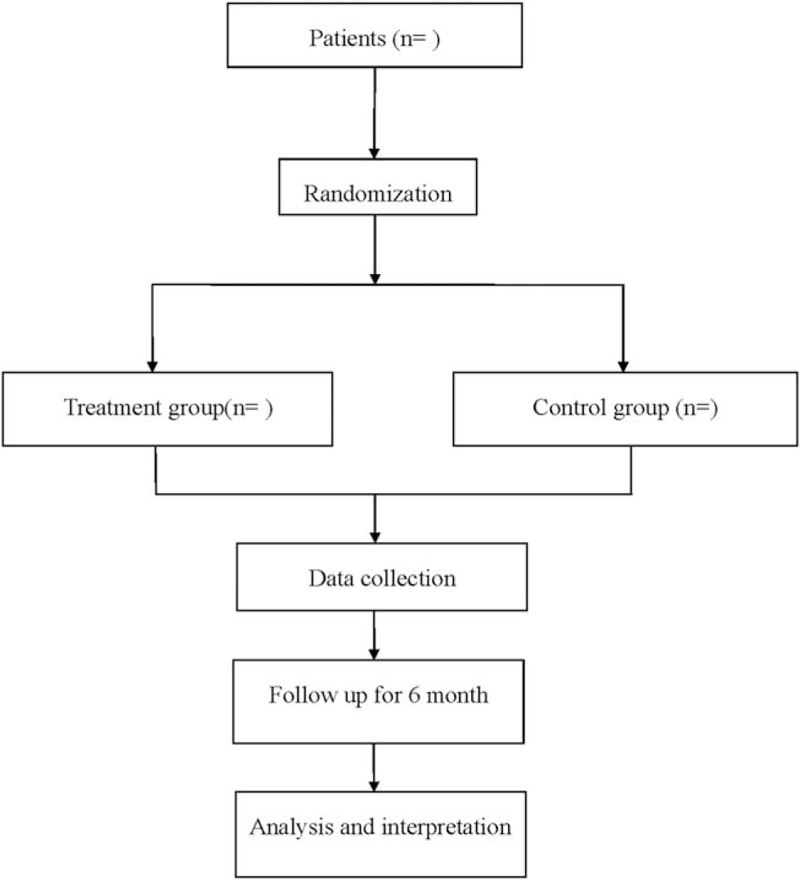
Flow diagram of the study.

This is a single-center, randomized, controlled clinical trial (CHiCTR), approved by the Ethics Committee of traditional Chinese medicine, and it will be carried out in accordance with the Declaration of Helsinki. A flowchart of this study is shown in Figure 1. The study conforms to the Standard Protocol Recommendations for Interventional Trials 2013 Statement,^[13]^ and the results will be reported according to the CONSORT Statement extension for trials.^[14]^

The protocol will be in accordance with the Helsinki Declaration and approved by the Clinical Research Ethics Committee of our hospital. This experiment has been registered in the open science framework (OSF) (registration number: DOI 10.17605/OSF.IO/ME6Y5). Before randomization, all patients will sign a written informed consent, and they can freely choose whether to continue the trial at any time.

### 2.2 Participants

Participants will be mainly recruited from inpatients in Hospital of traditional Chinese medicine. Previous report^[15]^ showed that medication treatment against HICH could reduce intracerebral hemorrhage (ICH) volume in 55% in 7 days. According to our preliminary study, a 7-day-reduction of ICH volume is 82%, and taking *α* = 0.05 and *β* = 0.2, a sample size of 38 will be needed. Considering the loss to follow-up, 46 patients will be ultimately recruited.

Inclusion criteria are:

(1) diagnosed with HICH^[16]^;
(2) aged 18 to 75 years old;
(3) with 20 to 40 bleeding volume;
(4) Glasgow coma scale score of 8 to 12;
(5) written informed consent.

Exclusion criteria are:

(1) severe organ dysfunctions or clotting disorders;
(2) complicated with ischemic stroke, or nervous system tumors, or mental illness.

### 2.3 Randomization and blinding

To minimize selection bias, patients will be randomly divided into treatment group and control group in a 1:1 ratio using the random number generator in Microsoft Excel. The patients and the neurosurgeons will not be blinded, and an independent researcher of no interest with the study will analyze the data.

### 2.4 Interventions

Stereotactic soft channel minimally invasive intracranial hematoma puncture and drainage treatment will be performed for patients in the treatment group with the following procedure:

(1) Locate the patient's puncture point, puncture angle, and catheter depth based on the level displayed on the preoperative computed tomography.
(2) Mark the puncture point perform local skin anesthesia.
(3) Make a 3 cm coronal linear skin incision lateral to the midline over the frontal area, and penetrate the skull with an electric drill. Insert the No. 12 soft channel drainage tube into the hematoma cavity according to the pre-designed direction and depth, pull out the guide core, and then drainage gently with a 5 mL syringe.
(4) Perform liquefaction and drainage about 4 hours after the puncture with urokinase.
(5) Extubate after the hematoma is basically removed after computed tomography scan.

Conservative treatment will be applied to patients in control group, including controlling blood pressure and glucose, anti-infection, relieving cerebral edema, fluid infusion, nutritional support, and symptomatic treatment.

### 2.5 Outcome variables

Vital signs and vital organs functions will be closely monitored. The primary outcomes are ICH volume reduction on day 0, 1, 3, and 7, coma condition measured by Glasgow coma scale and neurological function by National Institutes of Health Stroke Scale at week 0, 1, 2, 4, and then every month for 6 month. Secondary outcomes are activity of daily living at week 0, 1, 2, 4, and then every month for 6 month. Length of hospital stay and complications, and all possible adverse events will be recorded.

### 2.6 Statistical methods

All statistical analyses will be conducted by SPSS version 22 (IBM, Chicago, IL). Measurement data will be described in mean and standard deviation or median and interquartile range, and categorical variables as number and percentage. Different statistic method will be applied according to different data type. *P* < .05 is considered statistically significant.

## 3 Discussion

HICH is a spontaneous cerebrovascular disease caused by the rupture of artery, vein or capillary in the brain.^[17,18]^ The hematoma can cause brain cell edema and increased intracranial pressure, and patients would suffer from neurological dysfunction such as decreased limb muscle strength and impaired consciousness. At present, reducing the disability as much as possible and improve the quality of life of patients are critical in the treatment of HICH. In recent years, the application of MIS to remove intracerebral hematomas is more helpful than conventional craniotomy in improving postoperative recovery, reducing postoperative complications, and raising survival rates. MIS offered promise as a means to maximize hematoma evacuation while minimizing damage to normal tissue. Therefore, we try to carry out a prospective trial to compare the difference of ICH volume reduction, neurological function, and activity of daily living between MIS treatment and medication treatment against HICH. We hope that our results will be helpful for the choice between MIS and conservative treatment when treating HICH patients.

## Author contributions

**Data curation:** Jiang Liu, Jing Cheng.

**Funding acquisition:** Zhengxin Wang.

**Investigation:** Hongjun Zhou.

**Resources:** Hongjun Zhou, Chunyan Deng.

**Software:** Chunyan Deng.

**Writing – original draft:** Jiang Liu, Jing Cheng.

**Writing – review and editing:** Jiang Liu, Zhengxin Wang.
